# Diversity of Groundwater Microbial Communities near Sludge Repositories with Different Types and Levels of Pollution

**DOI:** 10.3390/life15121854

**Published:** 2025-12-02

**Authors:** Nadezhda Popova, Alexey Safonov

**Affiliations:** Frumkin Institute of Physical Chemistry and Electrochemistry Russian Academy of Sciences (IPCE RAS), 31-4, Leninsky Prospect, 119071 Moscow, Russia; nm.popova.ipce.ras@gmail.com

**Keywords:** mining and ore-processing sludge repositories, groundwater complex pollution, core microbiome diversity, Principal component and correlation analysis

## Abstract

Multicomponent pollution of groundwater with nitrates and sulfates is a common issue associated with mining and ore-processing operations. This work presents the first large-scale comparative study of groundwater microbial communities from six geographically distant sites in the Russian Federation with varying levels of nitrate and sulfate pollution. Based on high-throughput 16S rRNA sequencing data and hydrochemical analysis, a statistically significant influence of the pollution type on the structural and functional diversity of the microbiome was established. Nitrates act as a stimulating factor, increasing alpha-diversity, while sulfates have an inhibitory effect. Principal component and correlation analysis revealed spatial grouping of samples according to the predominant pollution type. Microbiome representatives common to all sites under unpolluted conditions were identified: *Bacteroides*, *Iamia*, and *Paenibacillus*; and under high pollution levels: *Acidovorax*, *Pseudomonas*, *Sphingomonas*, *Acinetobacter*, and *Limnohabitans*. Based on the obtained data, it is concluded that representatives of these genera are the most promising and universal for isolation and use in bioremediation.

## 1. Introduction

The stability of ecosystems is fundamentally linked to the functional and phylogenetic diversity of their microbial communities, which drive the biogeochemical cycles responsible for the flux of bioessential elements through living matter [1]. Within anthropogenically impacted environments, the efficacy of these microbial biogeochemical processes becomes critical for maintaining ecological stability and intrinsic self-purification potential [2]. These processes are particularly important in oligotrophic ecosystems, such as wetlands and groundwater, which are characterized by low nutrient levels, scarce organic matter, and cold temperatures [3]. Due to the limitations, such systems are highly susceptible to pollution and often unable to recover autonomously [4,5]. A prime example is groundwater affected by surface storage of chemical waste from metal ore mining and processing [6,7]. The widespread use of nitric and sulfuric acids in these industries leads to significant leaching of soluble pollutants—including nitrate, sulfate, and carbonate anions, as well as iron and other metals—into adjacent aquifers [8,9,10,11]. Wastes from uranium mining are of particular concern due to the presence of highly toxic uranyl ions [8]. Such multicomponent groundwater pollution is a common challenge, typically addressed through engineered solutions, most commonly through the installation of reactive or low-permeability (e.g., slurry wall) subsurface barriers [12,13,14,15,16].

The principle of in situ bioremediation relies on harnessing the innate biogeochemical capabilities of indigenous microbial communities. This promising and cost-effective approach involves stimulating natural nitrogen and sulfur cycles through the addition of organic substrates and phosphorus sources [17,18]. Such stimulation promotes the reduction of nitrate to molecular nitrogen via denitrification, as well as the conversion of sulfate to sulfide through sulfate reduction, leading to the precipitation of mineral phases. Notably, these microbial processes also facilitate the immobilization of uranium and other toxic metals, both through the formation of stable mineral phases [17,19] and direct enzymatic reduction [20,21,22]. Theoretical frameworks for applying in situ bioremediation to radionuclide-polluted aquifers are well-established [23,24,25]. Furthermore, field studies conducted at radiochemical sites in the USA and China [26,27,28,29] have validated this strategy for the comprehensive removal of nitrogen compounds and organic pollutants from groundwater. The success of in situ bioremediation is largely governed by the physiological diversity and abundance of the native microbiota [30]. Consequently, identifying microbial taxa that are widespread across polluted sites with varying pollution profiles is crucial for developing effective biopreparations, which can be administered alongside organic substrates to enhance remediation.

Hence, characterizing microbial diversity in polluted environments is essential for a dual purpose: evaluating the ecosystem’s intrinsic self-purification potential and determining the feasibility of in situ bioremediation. This is particularly relevant for groundwater ecosystems, which present unique challenges for anaerobic remediation processes due to their characteristic low levels of organic matter and phosphorus, consistently low temperatures (typically 6–10 °C), and transitional redox conditions. Additionally, the high salinity often encountered in polluted plumes necessitates the activity of halotolerant or halophilic bacteria. A critical functional requirement is the presence of a complete denitrifying community capable of reducing nitrate fully to molecular nitrogen, thereby preventing the accumulation of toxic intermediates like nitrite or ammonium, which could otherwise exacerbate system toxicity and undermine the bioremediation effort.

This study investigates the microbial diversity in groundwater systems at six geographically distinct locations across the Russian Federation, all of which are subject to comparable pollution profiles dominated by nitrates, sulfates, and ammonium. The sites encompass major industrial facilities, including the JSC Angarsk Electrochemical Plant (AECC) (Irkutsk Region, Russia), JSC Chepetsk Mechanical Plant (ChMP) (Udmurt Republic, Russia), and JSC Zelenogorsk Electrolysis and Chemical Plant (ECP) (Krasnoyarsk Krai, Russia), PJSC Novosibirsk Chemical Concentrates Plant (NCCP) (Novosibirsk region, Russia), and the Sublimation plant (SP) (Tomsk region, Russia), which are involved in processing polymetallic ores and purifying various metals, including uranium. The Siberian Chemical Combine (SCC) (Tomsk region), also included, specializes in reprocessing irradiated nuclear fuel. A common feature of these sites is the long-term surface storage of nitric and sulfuric acid wastes, neutralized with calcium hydroxide, in slurry reservoirs. After decades of operation (exceeding 50 years), the integrity of these reservoirs’ liners has been compromised, resulting in the leaching of soluble components—notably nitrates, sulfates, and carbonates—into the underlying groundwater. Detailed site characteristics are available in previous publications [17,31,32].

The current status of these sites varies; for instance, an impermeable barrier utilizing liquid glass technology has been installed in the area of the B2 basin (SCC), which was decommissioned in 2012, while the other facilities remain operational. Promisingly, pilot-scale trials of in situ bioremediation near the ChMP and B2 repositories demonstrated successful nitrate removal. This was achieved by stimulating the indigenous microbial community with a mixture of sugar and whey [33,34]. These trials identified biological nitrate removal as a critical initial step, as it drives the reduction of the redox potential, thereby establishing the anoxic conditions required for subsequent sulfate reduction. Ammonium elimination, meanwhile, can occur aerobically via nitrifying bacteria or anaerobically through the anammox process. Previous microbiological assessments at the AECC, NCCP, and ChMP sites revealed that highly polluted zones support physiologically diverse consortia comprising denitrifiers, nitrifiers, and anammox bacteria, underscoring the potential for stimulating these native communities to remediate nitrate pollution [35,36]. Furthermore, integrated monitoring has shown that microbial activity can induce geochemical conditions favorable for the precipitation and immobilization of various metals within biogenic mineral sediments [19].

The primary objective of this research was to evaluate how groundwater microbial diversity is influenced by physicochemical conditions across six sites impacted by nitrate-sulfate slurry reservoirs, and to identify consistent successional patterns in community structure in relation to the composition and magnitude of multicomponent pollution. From an applied perspective, a key goal was to identify the most prevalent and ubiquitous microbial taxa across the studied sites as potential candidates for developing specialized bioremediation consortia.

## 2. Materials and Methods

**Figure 1:**
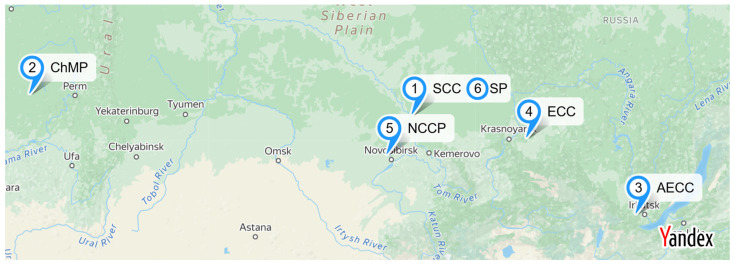
Sampling map of 6 sites (generated via https://yandex.ru/map-constructor, accessed on 10 October 2025).

In this work, 18 groundwater samples collected from 6 sites from depths of 10–20 m were used for comparison. Samples were taken from zones located near the waste reservoir (10–30 m), characterized by a high pollution level; at an intermediate distance (30–100 m); and in zones not affected by the reservoir (background samples). Sample labeling is provided in Table 1. The geography of sampling differed significantly (Figure 1) and included the central part of the Russian Federation (Glazov city, Udmurt Republic, 58°08′ N 52°40′ E), Novosibirsk Oblast (Novosibirsk, Russia, 55°01′ N. 82°55′ E), Krasnoyarsk Krai (Seversk, 56°29′19″ N. 84°57′08″ E, Zelenogorsk, 56°06′00″ N 94°35′00″ E), and Irkutsk Oblast (Angarsk, 52°34′ N 103°55′ E).

### 2.1. Sampling

Following a 15–30 min fluid circulation to purge the wells, groundwater was sampled from 10–20 m depth. Samples for cation and anion analysis were collected in 200 mL bottles without fixation in triplicate. For U and Fe analysis samples were fixed by high grade pure HNO_3_ to pH 1 in triplicate. For molecular biology, 2 L of water (in duplicate) were fixed with ethanol (1:1 *v*/*v*) and filtered through 0.22-μm membranes. For microbiological cultivation on nutrient media, samples were collected in 100 mL sterile glass bottles in triplicate. Prior to inoculation, the samples were stored in a refrigerator at 4 °C for 3–5 days. Temperature, pH, and *Eh* were measured on-site immediately after sampling.

For this study, the most significant components in terms of concentration, as well as elements with notable toxicity (e.g., uranium), were selected for analysis. The analysis of the samples did not detect organic pollutants (e.g., hydrocarbons, POPs) or radionuclides other than uranium, as the sites in question are associated with metal and uranium ore processing enterprises. The concentrations of heavy metals (Zn, V, Mo, Cr) were low in the proximal zone and fell below the detection limit in areas with intermediate pollution levels. Consequently, data on their concentrations, along with any potential contribution to biogeochemical processes, were excluded from the present manuscript.

The level of pollution used in the statistical analysis was determined both by the location of the wells and by parameters such as total dissolved solids (TDS), nitrate ion concentration, and the concentrations of other major components. The study utilized existing monitoring wells and hydrogeological data provided by the industrial sites. No specialized drilling was conducted for this particular investigation.

### 2.2. Analytical Methods

#### 2.2.1. Hydrochemical Analysis

The uranium and iron concentrations in the water samples were analyzed immediately after sample collection and filtration through a 0.45-µm glass filter by Inductively Coupled Plasma Mass Spectrometry (ICP-MS) X Series2 (Thermo Fisher Scientific, Waltham, MA, USA, https://www.fishersci.com/shop/products/icap-6500duoview-icp-oes-spect/NC1982295, accessed on 11.09.2025).

Anion and cation concentrations were measured using a capillary gel electrophoresis system (Capel-105M, LUMEX Instruments, Saint Petersburg, Russia, https://www.lumexinstruments.com/catalog/capillary-electrophoresis/capel-105m.php, accessed on 09.08.2025).

The Eh and pH values were determined using an ANION-4100 pH meter/ionomer (Novosibirsk, Russia, http://www.anion.nsk.su/, accessed on 02.08.2025) with an electrode combination vs. Ag/AgCl electrode.

Total organic carbon in water was measured using an Elementar Vario EL III CHN analyzer (Elementar Analysensysteme GmbH, Langenselbold, Germany).

#### 2.2.2. Molecular Analysis

DNA was extracted from groundwater samples fixed with ethanol by filtering through membrane filters with a pore diameter of 0.22 µm (Millipore, Merck, Darmstadt, Germany). The sediment from the filter surface was washed off with a solution containing 0.15 M NaCl and 0.1 M Na_2_EDTA (pH 8.0). The Pure LinkTM Microbiome DNA Purification KIT (ThermoFisher Scientific, Waltham, MA, USA) was used for DNA extraction according to the manufacturer’s recommendations.

High-Throughput Sequencing of V4 Fragments of the 16S rRNA Gene: To determine the composition of microbial communities in the biofilms, the hypervariable V4 region of the 16S rRNA gene was amplified. Amplification was performed in a mixture containing 5 µL of each primer (6 µM concentration), 5 µL of DNA solution, and 15 µL of PCR mix (1 U polymerase, 0.2 mM of each dNTP, 2.5 mM Mg^2+^), using primers Pro341F-Pro805R [37]. Each sample was amplified in triplicate, then the replicates were pooled together and purified by electrophoresis in a 2% agarose gel using a kit for extraction and purification of PCR products from the gel (Eurogen, Moscow, Russia). Sequencing was performed using the MiSeq platform (Illumina, San Diego, CA, USA) and the MiSeq V3 (600 cycles) reagent kit (Illumina, San Diego, CA, USA) according to the manufacturer’s recommendations. Gene libraries were created using dual barcoding, as described previously [38].

Bacterial community libraries were created using the online SILVA resource (https://www.arb-silva.de/ngs/, accessed on 24.09.2025). Raw reads were processed and assembled as previously described [39]. Contigs were binned and refined, MAGs were reassembled by BASALT v1.1.0 [40]. MAGs were analyzed by METABOLIC v4.0 [41]. Adapters and primers were trimmed with cutadapt v2.8 [42], quality filtering was done by Trimmomatic v0.36 [43]. Demultiplexing was done by deML v1.1.4 [44]. Chimeric sequences were removed and the amplicon sequence variant (ASV) table was constructed using Dada2 v1.26.0 package [45] and the SILVA 138.2 database [45]

Determination of the number of main physiological bacterial groups (aerobic organotrophic and anaerobic denitrifying bacteria, ferric iron-reducing, sulfur-reducing, nitrite and ammonium oxidizing) was determined by inoculating 10-fold dilutions of groundwater samples (in triplicate) into liquid nutrient media. Microbial numbers were calculated according to the most probable number technique using McCready tables. Media content is described in [34,46] and provided in Table A1.

#### 2.2.3. Statistical Methods

Data statistical processing was performed in the Origin 2024 program using built-in tools. A one-way, two-way analysis of variance (ANOVA were further used to test the significance level of the hydrochemical parameters influence. The iVikodak online platform (https://web.rniapps.net/iVikodak/, accessed on 24 September 2025) [47] was used to predict the functional characteristics of bacterial communities. Biodiversity indices were calculated via Past4 statistical software (https://palaeo-electronica.org/2001_1/past/pastprog/univar.html, accessed on 24 September 2025). Venn diagrams were constructed using the online resource E-Venn (https://www.bic.ac.cn/test/venn/#/, accessed on 24 September 2025) [48].

## 3. Results

### 3.1. Comparison of Sample Chemical Composition

The hydrogeochemical analysis and diversity indices of the collected groundwater samples are summarized in Table A1. All objects differ statistically significantly in hydrogeochemical and microbiological parameters (*p* < 0.001, Table A3, Table A4 and Table A5). Background samples exhibited reducing redox potentials, low overall mineralization, and diminished concentrations of organic matter and bioessential elements. Their anion composition was dominated by bicarbonates and sulfates, which are characteristic of natural groundwater systems. Iron concentrations in these pristine zones varied across horizons, ranging from hundreds of µg/L to several mg/L. In contrast, samples influenced by technogenic impact showed marked elevation in the concentrations of nitrates, sulfates, bicarbonates, ammonium, calcium, sodium, and iron. Notably, iron content reached levels as high as 17 mg/L in heavily polluted samples. Uranium was detected in the range of 0.4–2 mg/L in polluted groundwater, substantially exceeding the maximum permissible concentration.

**Figure 2:**
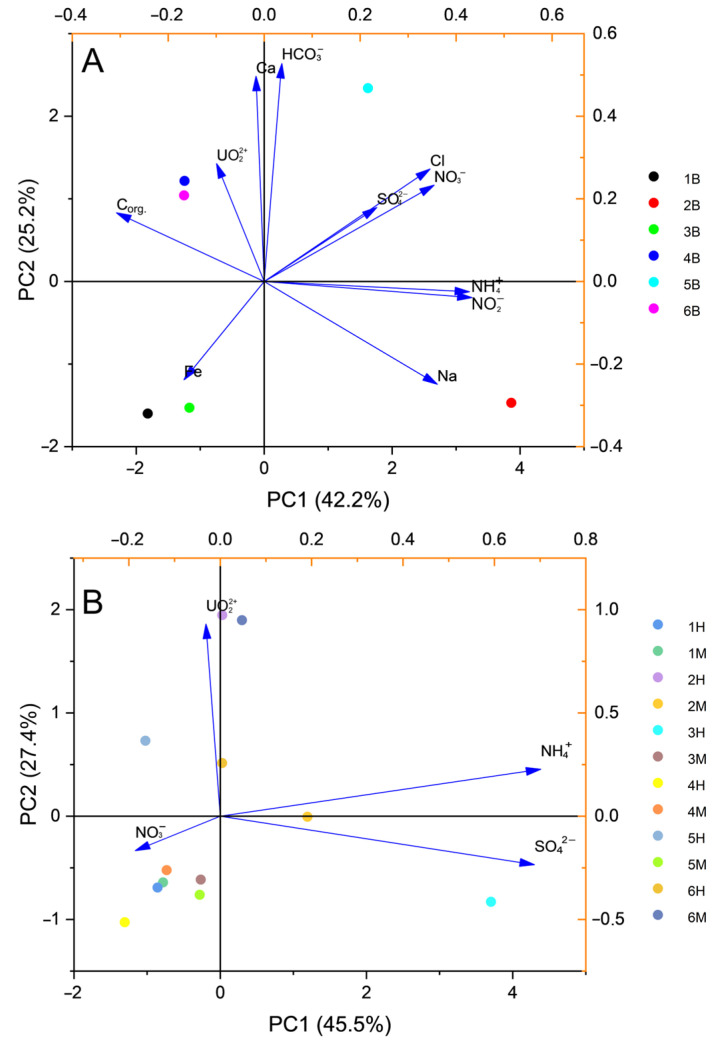
Principal component analysis (PCA) of the chemical composition of samples from background (**A**) and polluted zones (**B**).

Multivariate analysis (Principal component analysis, PCA) based on solute concentrations revealed distinct sample clustering (Figure 2A,B). Background samples from Sites 1, 3, 4, and 6 grouped together and were associated with higher organic carbon content. A discernible correlation was also observed between the anions of nitric and sulfuric acids, alongside ammonium and carbonate ions, specifically in groundwater from Sites 2 and 5. Further analysis focusing on wells with medium and high pollution levels (Figure 2B) showed a clear clustering of samples 1M, 1H, 3M, 4M, 4H, 5M, and 5H along Principal Component 1 (PC1), indicating a strong common influence of nitrate pollution. Sample 3H was uniquely characterized by a pronounced positive loading of sulfate and ammonium ions. Furthermore, samples 2H, 2M, and 6M were distinguished along PC2, primarily due to their significant uranium pollution.

### 3.2. Characterization of Biodiversity in Background and Polluted Samples

#### 3.2.1. Microbial Diversity Gradients Relative to Pollution Sources

**Figure 3:**
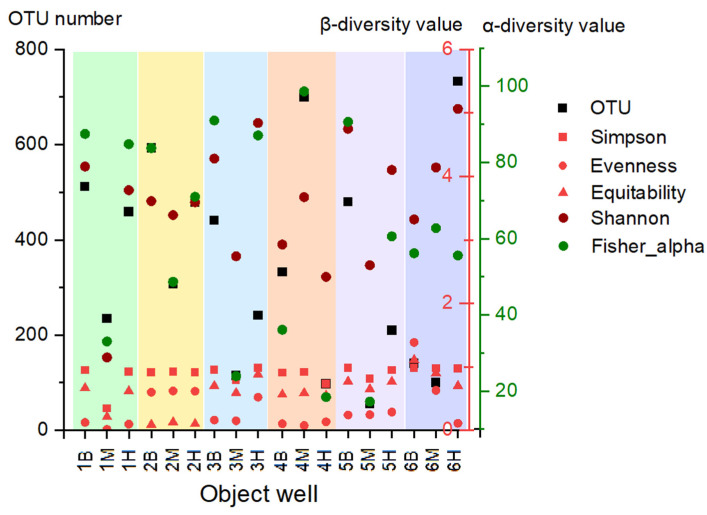
Biodiversity indices of microbial communities from sites with different pollution levels. Green—Site 1, yellow—Site 2, blue—Site 3, orange—Site 4, violet—Site 5, purple—Site 6.

Analysis of biodiversity parameters revealed substantial heterogeneity in microbial communities across all sampling zones, encompassing background areas and those with medium to high pollution levels (Figure 3). The highest levels of biodiversity, based on both OTU counts and diversity indices, were generally associated with background wells (1B, 2B, 3B, 5B). In contrast, the most taxonomically impoverished communities (OTUs < 150) were identified in several aquifers subject to medium and high pollution pressure, specifically 3M, 4H, 5M, 6M, and 6H. An interesting exception was community 1M, which exhibited a high taxonomic richness relative to other polluted sites; however, the strong dominance of specific groups within this community led to a reduction in its overall diversity indices, including Simpson, Shannon, Evenness, and Fisher. However, in samples from sites 3 and 6, high diversity was found in zones with medium and high pollution levels (3M, 6H). Characterization of alpha-diversity (Fisher index) demonstrated high index values in samples from background wells and wells with medium pollution levels (1–2M, 4M, 6M), with the exception of sites 3M and 5M.

The response of microbial diversity to increasing pollution pressure varied substantially among the sites. Sites 1, 2, 3 and 5 exhibited a diversity minimum at intermediate pollution levels. Conversely, a positive relationship was found at Site 6, where diversity increased with the pollution gradient, while Site 4 showed a similar peak in diversity at a medium pollution level.

#### 3.2.2. Phylogenetic Composition of Background Samples

**Figure 4:**
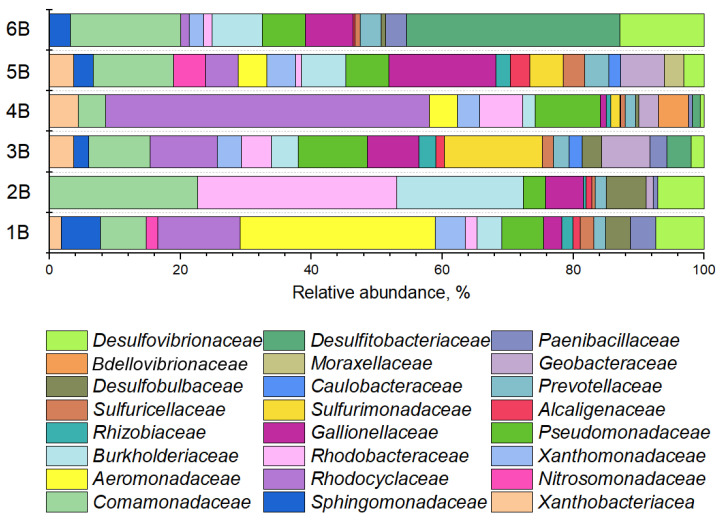
Diversity of groundwater background sample microbiomes at the family level.

In the composition of microbial communities from background samples (Figure 4), bacteria typical of oligotrophic habitats involved in iron and sulfur cycles were found, as well as organotrophic bacteria from the families *Comamonadaceae, Rhodocyclaceae, Rhodobacteraceae, Burkholderiaceae, Pseudomonadaceae, Gallionellaceae, Prevotellaceae, Desulfovibrionaceae*.

The phylogenetic structure of the background groundwater communities revealed distinct site-specific profiles, which were determined by the unique geochemical and geological parameters of the aquifers. In sample 1B, the microbial community was dominated by members of the family *Aeromonadaceae*. These mesophilic bacteria are recognized for their metabolic versatility, being capable of heterotrophic nitrification and aerobic denitrification under elevated pH conditions, with some strains also demonstrating Fe(III) reduction capabilities [49]. A different composition was observed in community 2B, where a consortium of *Comamonadaceae*, *Rhodobacteraceae*, and *Burkholderiaceae* collectively accounted for over 70% of the relative abundance. Taxa within these families are known for their central roles in nitrogen and sulfur cycling and possess the genetic potential for heavy metal reduction [50,51,52]. In contrast, community 3B lacked a strongly dominant taxon, with the most abundant group, *Sulfurimonadaceae*—implicated in sulfur-driven autotrophic denitrification [53]—comprising just over 10% of the population. A markedly different structure was found in community 4B, which was characterized by a pronounced dominance of *Rhodocyclaceae*, representing approximately half of the entire community. Meanwhile, community 5B was primarily composed of *Comamonadaceae* and iron-oxidizing bacteria from the family *Gallionellaceae*, which together constituted nearly 30% of its composition. Finally, the background community 6B was dominated by sulfate-reducing bacteria affiliated with the family *Desulfitobacteriaceae* [54].

**Figure 5:**
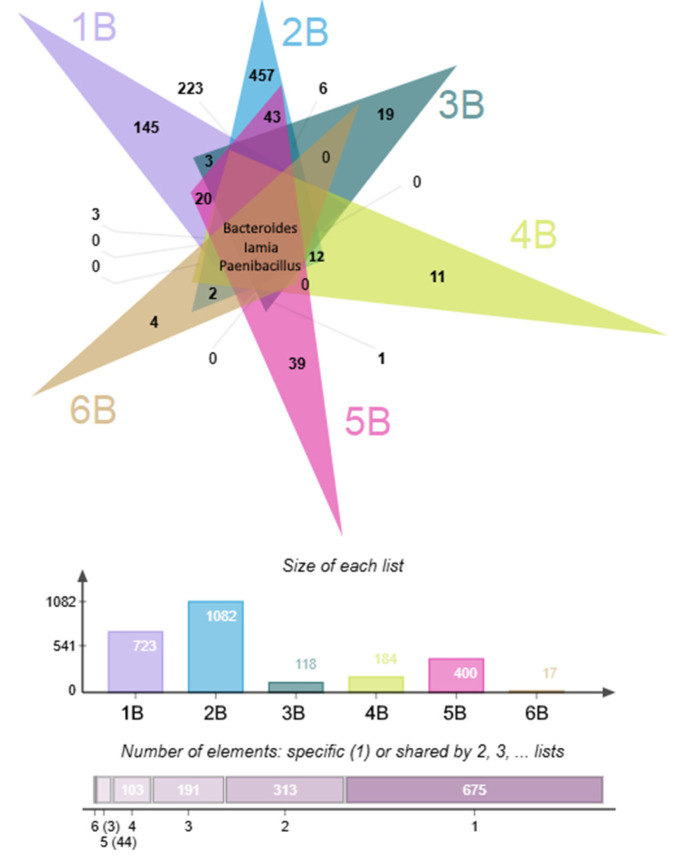
Venn diagrams showing the overlap of the background bacterial communities (core microbiome), the total amount of present genera in each community, and the number of shared genera between 6, 5, 4, 3, and 2 communities and genera belonging to a single community.

A comparative analysis of all background communities, visualized via Venn diagram (Figure 5), identified a core set of genera ubiquitous across all sites, including *Bacteroides*, *Iamia*, and *Paenibacillus*. Beyond this universal core, significant sharing of taxa was observed, with 44 genera common to five out of the six sites and 103 genera shared among four sites, indicating a considerable degree of phylogenetic overlap within these groundwater ecosystems.

#### 3.2.3. Phylogenetic Composition of Polluted Samples

**Figure 6:**
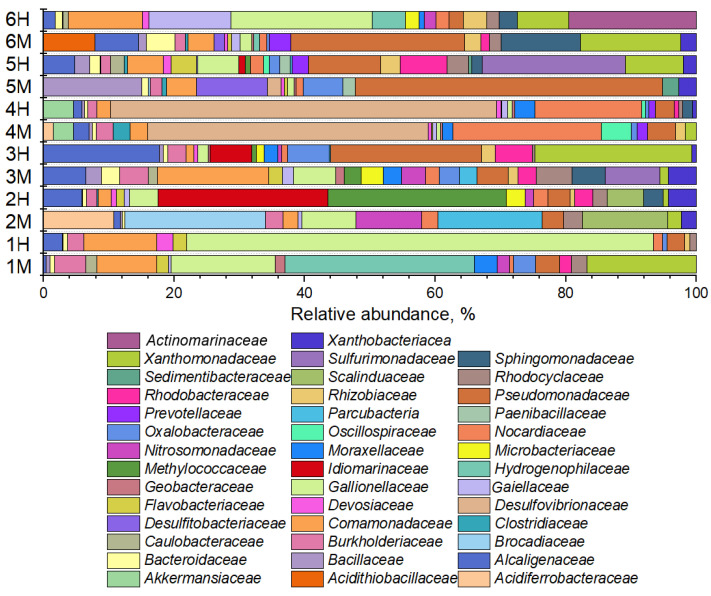
Phylogenetic composition of contaminated groundwater microbiomes.

The phylogenetic analysis of polluted groundwater samples revealed the recurrent presence of several bacterial families, including *Alcaligenaceae, Bacillaceae, Bacteroidaceae, Burkholderiaceae, Comamonadaceae, Gaiellaceae, Gallionellaceae, Nocardiaceae, Pseudomonadaceae, Rhodocyclaceae, Xanthomonadaceae* and *Xanthobacteriacea* (Figure 6). 

At Site 1, characterized by nitrate-dominated pollution, the communities were dominated by *Gallionellaceae* (increasing from 17.6% in 1M to 83% in 1H) and *Comamonadaceae* (9% in 1M to 13.7% in 1H). The moderately contaminated sample 1M also showed significant abundances of *Hydrogenophilaceae* (21.1%) and *Xanthomonadaceae* (16.3%). Notably, samples from Site 2, subject to high ammonium pollution, harbored specialized microorganisms, including anammox bacteria from the families *Brocadiaceae* (11.2% in 2M) and *Scalinduaceae* (16.6% in 2M, 7.4% in 2H). These samples also contained acidophilic iron- and sulfur-oxidizing *Acidiferrobacteraceae* (11.2% in 2M) [55] and nitrate-reducing *Idiomarinaceae* (27.7% in 2H) [56]. The community under mixed nitrate-sulfate pollution at Site 3 (sample 3M) was composed of *Comamonadaceae* (15%) and *Sulfurimonadaceae* (7.6%). Under more severe multicomponent pollution (3H), the community shifted towards denitrifying *Alcaligenaceae* (18.6%) [57], with dominant representation from *Pseudomonadaceae* (24.2%) and *Xanthomonadaceae* (25.1%). A unique pattern was observed at Site 4, where sulfate-reducing *Desulfovibrionaceae* were exclusively detected under extreme nitrate pollution (36.1% in 4M, 48.8% in 4H), co-occurring with *Nocardiaceae* (19.1% in 4M, 13.5% in 4H). At Site 5, subject to nitrate-sulfate pollution, the community in sample 5M was dominated by *Pseudomonadaceae* (40.2%), *Bacillaceae* (12.8%), and *Desulfitobacteriaceae* (9.2%). Under higher pollution (5H), the community transitioned to dominance by *Sphingomonadaceae* (22%), *Pseudomonadaceae* (11%), and *Xanthomonadaceae* (8.9%). Finally, at Site 6 with multicomponent pollution including uranium, the community in sample 6M comprised *Pseudomonadaceae* (25.5%), *Sphingomonadaceae* (11.7%), *Xanthomonadaceae* (14.7%), and *Acidithiobacillaceae* (7.5%). In the more polluted area, the community shifted toward *Gallionellaceae* (21.8%), *Comamonadaceae* (11.4%), *Gaiellaceae* (12.5%), and *Xanthomonadaceae* (8%).

**Figure 7:**
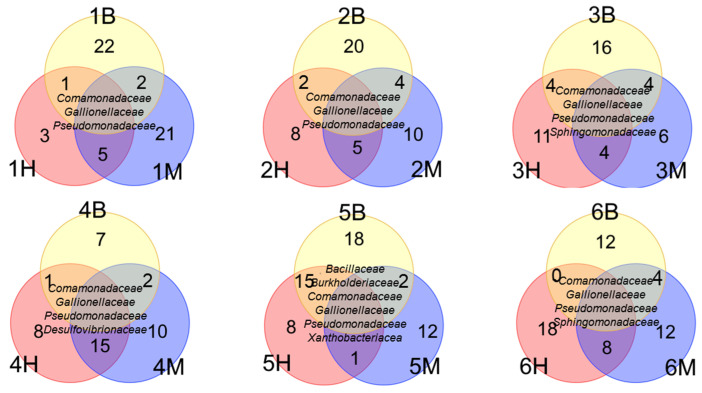
Shared phylogenetic features across polluted sites. Venn diagram illustrating the number of common (inner sector) and unique (outer sector) bacterial families among sampling sites (OTU > 1%).

**Figure 8:**
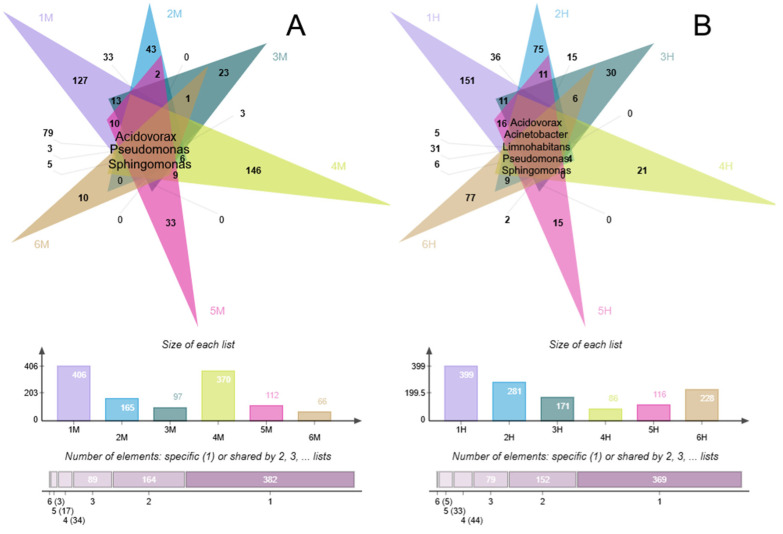
Venn diagrams showing the overlap of the bacterial communities with moderate pollution (**A**) and heavy pollution (**B**), the total amount of present genera in each community, and the number of shared genera between 6, 5, 4, 3, and 2 communities and genera belonging to a single community.

Comparative analysis revealed a conserved core of bacterial families across all polluted sites, consisting of *Comamonadaceae*, *Gallionellaceae*, and *Pseudomonadaceae* (Figure 7). Additional shared phylogenetic features included the presence of *Sphingomonadaceae* in Sites 3 and 6, and the consistent detection of *Desulfovibrionaceae* across all samples from Site 4. The communities from Site 5 showed the highest internal similarity, sharing six common taxa. At the genus level, we identified a universal core of *Acidovorax*, *Pseudomonas*, and *Sphingomonas* across all polluted samples (Figure 8). Furthermore, all highly polluted communities specifically contained *Acinetobacter* and *Limnohabitans*.

The degree of phylogenetic overlap varied substantially with pollution intensity. Communities from moderately polluted samples showed significantly lower similarity (17 genera shared among 5 sites; 34 among 4 sites) compared to background communities (44 and 103 genera, respectively). In contrast, heavily polluted samples demonstrated greater phylogenetic convergence (33 genera shared among 5 sites; 44 among 4 sites), indicating consistent selection for specific bacterial lineages under severe pollution pressure.

### 3.3. Metabolic Potential of Microbial Communities

**Figure A1:**
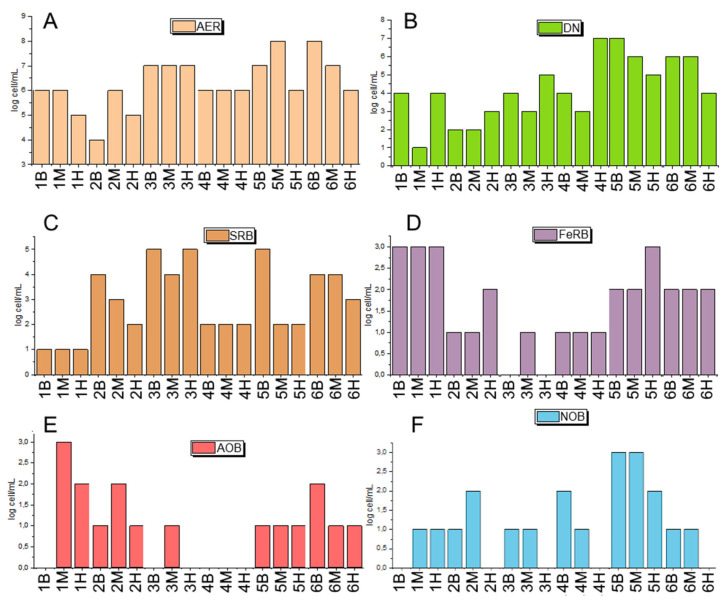
Log cell counts of the main functional groups of microorganisms in groundwater samples. (**A**) AER—aerobic organotrophic bacteria, (**B**) DN—denitrifying, (**C**) SRB—sulfate-reducing, (**D**) FeRB—iron-reducing microorganisms, (**E**) AOB—nitrifying ammonium to nitrite, (**F**) NOB—nitrifying nitrite to nitrate.

Cultivation-based analysis of major physiological groups revealed distinct patterns across the pollution gradient (Figure A1). Denitrifying and aerobic bacteria showed increased abundance in moderately polluted zones compared to background areas. Quantification of denitrifying microorganisms indicated consistently high levels (>1000 cells/mL) across most samples (1B, 1H, 3B, 3H, 4B, 4H, 5B–H, 6B–H), with peak abundances observed in samples 4H, 5M, and 6M. Sulfate-reducing microorganisms reached their maximum densities in the most heavily polluted zones, while iron-reducing bacteria remained relatively scarce (<100 cells/mL), with elevated counts detected only in communities 1B–H and 5H.

**Figure A2:**
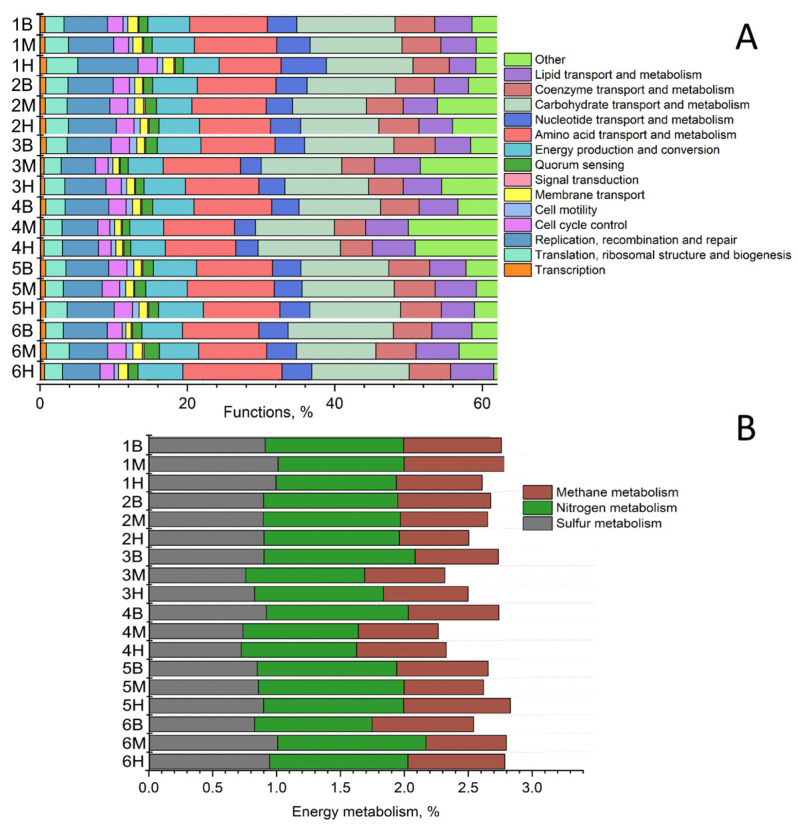
Metabolic pathway analysis via IVikodak global (**A**) and local mapper (**B**), associated with KEGG database.

Functional potential analysis using iVikodak with the KEGG database revealed systematic shifts in metabolic capabilities between polluted and background zones across multiple hierarchy levels. At KEGG Level 2 (Figure A2A), notable variability was observed in genetic information processing pathways (encompassing transcription, translation, and replication), with particularly elevated representation in communities 1H and 2H, suggesting enhanced cellular activity and growth in these polluted environments. Environmental information processing functions—including membrane transport, signal transduction, and quorum sensing—were most prominent in communities 2M, 6M, and 3B. Membrane transport capabilities were especially well-represented across communities from site 1 (1B–H) and in samples 2M, 6M, 6H, 3H, and 5H, indicating robust capacity for nutrient uptake and environmental interaction. Energy metabolism genes displayed a non-linear response to contamination, with elevated levels in highly polluted samples relative to background, but reduced representation in moderately polluted zones. Communities from site 1 (1B–H) constituted an exception to this pattern, maintaining elevated energy metabolism even at intermediate pollution levels.

At KEGG Level 3 (Figure A2B), analysis of specific biogeochemical pathways revealed further functional specialization. Sulfur metabolism genes showed parallel abundance patterns in sites 2 and 5, while reaching minimum levels in communities 4M, 4H, 6B, and 3M. Maximum sulfur metabolic potential was observed in communities 1B–H and 6M. Nitrogen metabolism genes were most strongly represented in communities 1B, 3B, 3H, 4B, 6M, 6H, and across all samples from site 5 (5B–H). In contrast, nitrogen transformation capabilities were minimal in communities 4M, 4H, 6B, and 3M. Methane metabolism genes were uniformly low across all communities, indicating limited involvement of methanogenic or methanotrophic processes in these ecosystems.

### 3.4. Correlation Analyses of Hydrochemical and Microbiological Parameters

**Figure A3:**
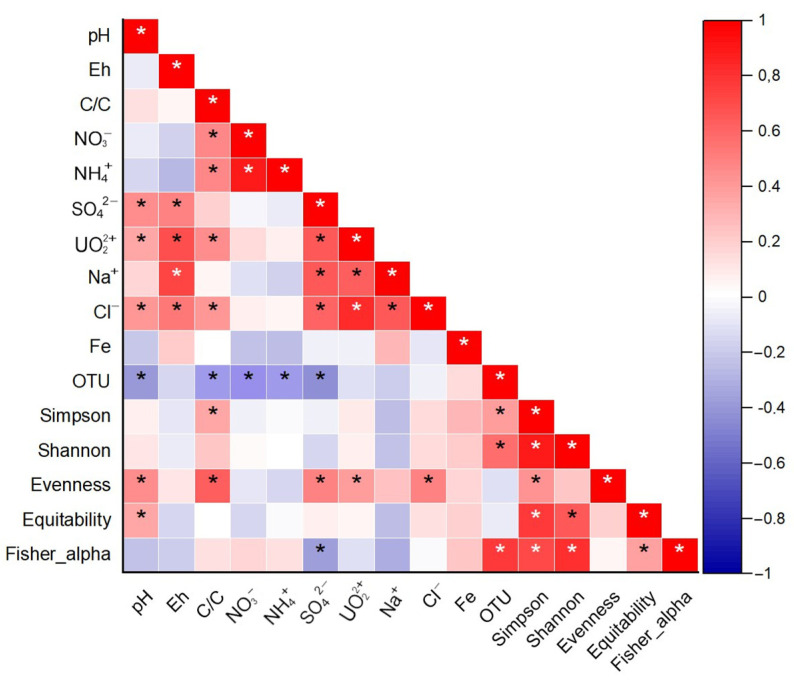
Correlation plot of hydrochemical and biodiversity relationships (* indicates *p* < 0.05 values).

Correlation analysis of hydrochemical and biodiversity relationships revealed systematic patterns in microbial community development under chemical pollution stress (Figure A3). The significant positive correlation between nitrogenous pollutants and OTU richness suggests a dual role for nitrogen compounds—acting both as stressors and as growth-stimulating nutrients that alleviate natural nitrogen limitation in oligotrophic groundwater systems. Notably, we observed a significant inverse relationship between microbial abundance and environmental pH, potentially reflecting both community adaptation to moderately acidic conditions created by pollutants and pH alterations resulting from microbial metabolite production. While nitrate pollution generally correlated with increased taxonomic richness, sulfate contamination demonstrated a strong negative association with OTU numbers. Analysis of community evenness revealed significant dependencies on pH, sulfate, uranyl, and chloride concentrations. Elevated levels of these ions appear to suppress taxonomic dominance, fostering more uniform community structures where no single taxon gains competitive advantage under chemical stress conditions.

**Figure A4:**
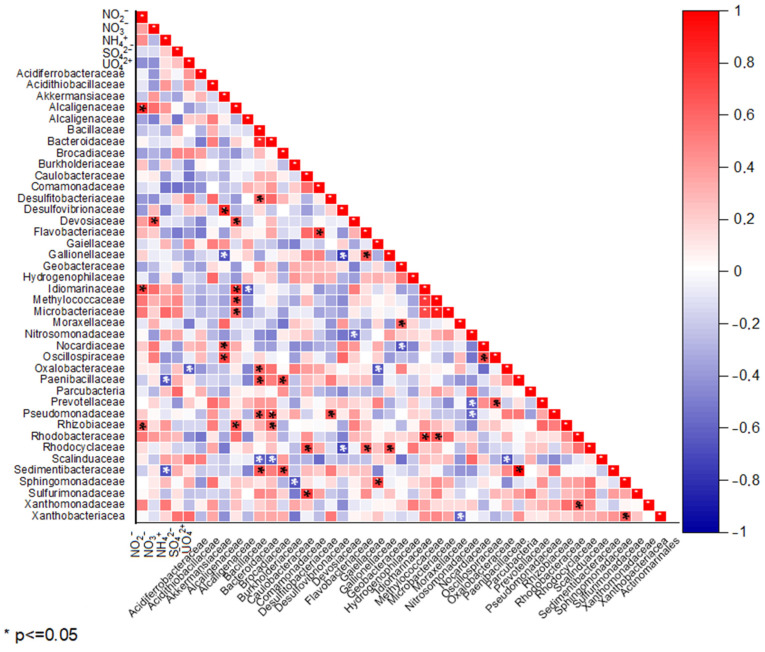
Spearman correlation diagram showing the presence of connections (* indicates *p* < 0.05) between hydrochemical parameters and phylogenetic diversity across all samples.

Correlation analysis between taxonomic composition and pollution levels identified specific family-pollutant relationships (Figure A4). Nitrate concentration showed significant positive correlations with *Devosiaceae* and *Pseudomonadaceae*, and strong associations with *Alcaligenaceae*, *Idiomarinaceae*, *Nocardiaceae*, *Oscillospiraceae*, *Rhodobacteriaceae*, and *Xanthomonadaceae*. Ammonium pollution correlated significantly with *Scalinduaceae* and showed strong positive relationships with *Xanthomonadaceae*, *Rhizobiaceae*, *Alcaligenaceae*, and *Microbacteriaceae*. Sulfate contamination demonstrated significant positive correlations with *Desulfovibrionaceae* and *Sulfurimonadaceae*, and notable associations with *Parcubacteria*, *Xanthomonadaceae*, *Brocadiaceae*, *Microbacteriaceae*, and *Idiomarinaceae*. While uranium showed no significant positive correlations, it influenced *Oxalobacteraceae* distribution and exhibited direct relationships with *Xanthobacteriaceae*, *Scalinduaceae*, *Rhodobacteriaceae*, *Galiellaceae*, *Desulfitobacteriaceae*, *Brocadiaceae*, and *Acidithiobacillaceae*.

**Figure 9:**
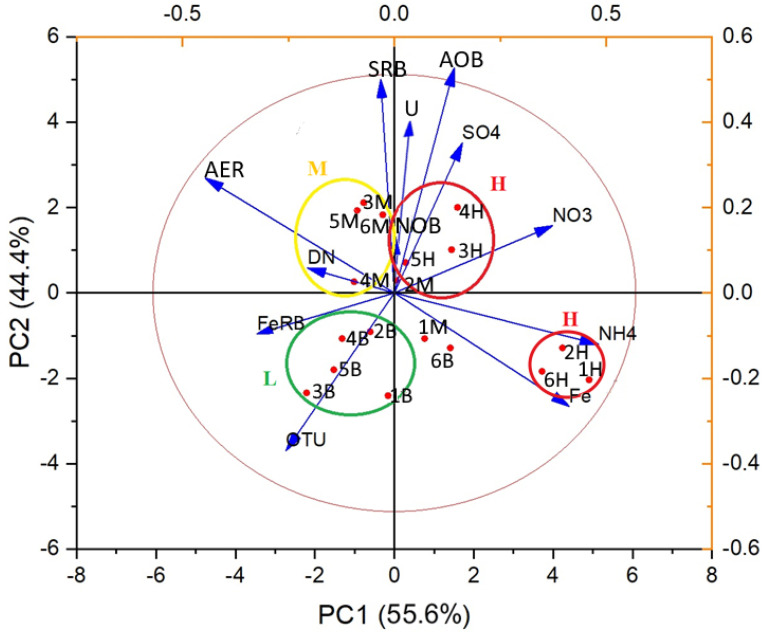
Principal component analysis (PCA) diagram of the sample distribution depending on the pollution level and bacterial physiological groups. Group L—low pollution (background samples): 1B, 2B, 3B, 4B, 5B; M—medium pollution level: 3M, 4M, 5M, 6M; H—high pollution level: 3H, 4H, 5H, 2M, 2H, 1H, 6H. Outliers: 1M, 6B. The arrows: AER—aerobic bacteria, SRB—sulfate-reducing bacteria, AOB—ammonium oxidizing bacteria, NOB—nitrite oxidizing bacteria, FeRB—ferric iron-reducing bacteria, DN—denitrifying bacteria.

Principal component analysis (PCA) clearly resolved spatial separation of microbial communities along contamination gradients (Figure 9). Background communities (Group L), clustering inversely to major pollutants along PC1, maintained higher OTU richness and were enriched with iron-cycling bacteria from *Geobacteraceae* and *Gallionellaceae*. Iron-oxidizing bacteria of the family *Gallionellaceae* (*Gallionella*, *Sideroxydans*) and iron-reducing bacteria of the family *Geobacteraceae* (*Geobacter*), under conditions of limited nutrient access, are capable of using clays as electron donors and humic substances as electron acceptors [58,59,60], suggesting their key role in anaerobic organic matter oxidation alongside aerobic organotrophs (*Acinetobacter*, *Acidovorax*, *Bacillus*, *Pseudomonas*, *Sphingopyxis*).

Ammonium-rich samples (1H, 2H, 6H) formed distinct clusters characterized by nitrogen-cycling specialists from *Gallionellaceae*, *Comamonadaceae*, *Hydrogenophilaceae*, *Xanthomonadaceae*, and *Scalinduaceae*, facilitating complete nitrogen compound processing from ammonium oxidation to removal. Samples with high sulfate-nitrate content clustered with nitrifying and sulfate-reducing bacteria, though 16S rRNA analysis detected *Desulfovibrionaceae* exclusively at Site 4, despite cultivation-based evidence of sulfate reduction at multiple sites. Sulfur-oxidizing bacteria (*Acidithiobacillaceae*, *Sulfurimonadaceae*) were widely distributed, with some *Gallionellaceae* and *Hydrogenophilaceae* representatives also capable of oxidizing reduced sulfur compounds [61,62,63,64].

Moderately polluted zones were dominated by aerobic heterotrophs and denitrifiers (*Pseudomonadaceae*, *Comamonadaceae*), thriving despite organic carbon limitation due to available nitrate, ammonium, sulfate, and bicarbonate.

## 4. Discussion

We examined groups of monitoring wells located along the flow path of the groundwater at varying distances from the 6 different U-containing sludge storage facilities: nearby wells (10–30 m) and intermediately located wells (30–50 m). In all cases, background samples were collected at a significant distance from the facilities (100–200 m) in areas situated away from the groundwater flow direction. The compositional analysis of the collected samples indicated that the multicomponent pollution was defined by elevated concentrations of nitrate, sulfate, ammonium, and bicarbonate ions. Uranium exhibited no significant correlations, which can be attributed to its relatively low environmental concentrations. Figure A3 presents a Spearman correlation diagram illustrating the relationship between microbial diversity and pollution types. Elevated nitrogen concentrations were strongly correlated with reduced microbial alpha-diversity (OTU count), showing significant negative correlations with nitrate (r = −0.68, *p* < 0.001), ammonium (r = −0.72, *p* < 0.001), and nitrite (r = −0.61, *p* = 0.002). Pollution type significantly shaped community response: nitrate mono-pollution (Sites 1, 4) reduced both biodiversity and nitrogen metabolism gene representation, while dual nitrate and ammonia contamination enhanced nitrogen cycle genes in energy metabolism (Figure A2). A potential explanation for this is that multicomponent pollution fostered two adaptation strategies—either diversifying nitrogen-cycling specialists (denitrifying bacteria, nitrifying bacteria, etc.) (3M, 3H) or selecting specialized dominants capable of utilizing both oxidized and reduced nitrogen forms (*Brocadiaceae, Scalinduaceae*) [36,65] (Sites 2, 3). Collectively, these factors may lead to a synergistic effect in the microbial community’s response to contamination. Furthermore, recent research has revealed potential couplings between the biogeochemical cycles of iron, sulfur, and other elements [50].

However, given that nitrate ions were the dominant contaminant across all sites, we posit that nitrate is the primary factor shaping the microbial community structure in the polluted zones and steering the direction of the dominant microbial processes. This is because denitrification is the most expected process in the investigated habitats under oxygen-limited conditions. This is supported by an observed increase in the abundance of denitrifying bacteria in areas with medium-level nitrate pollution at all sites, as well as in one site with high-level pollution (for example, Site 3). Further corroboration comes from increased diversity indices in zones with medium and even high levels of pollution (for example, Site 6), and by an enhanced contribution of nitrogen metabolism genes in communities 1B, 3B, 3H, 4B, 6M, 6H, and across all samples from site 5 (5B–H). In contrast, nitrogen transformation capabilities were minimal in communities 4M, 4H, 6B, and 3M (Figure 2A).

Correlation analysis between taxonomic composition and pollution levels identified specific family-pollutant relationships (Figure A4). Nitrate concentration showed significant positive correlations with *Devosiaceae* and *Pseudomonadaceae*, and strong associations with *Alcaligenaceae*, *Idiomarinaceae*, *Nocardiaceae*, *Oscillospiraceae*, *Rhodobacteriaceae*, and *Xanthomonadaceae*. Nitrate pollution consistently selected for bacteria performing dissimilatory nitrate reduction (*Acidovorax*, *Pseudomonas*) [66,67,68,69,70] and iron redox cycling (*Acidovorax*, *Pseudomonas*, *Gallionella*) [66,71]. *Acinetobacter*, a heterotrophic denitrifying bacteria [72,73,74], as well as *Azoarcus* and *Thauera* [70,75], also possess genes encoding nitrite reductases (nirS and nirK) and are commonly present in aquifers with nitrogen pollution. The remarkable functional resilience of these microbial communities to nitrate stress was further evidenced by the prevalence of diverse nitrogen-cycling microorganisms. 

However, it is known that an intensive denitrification process under high nitrate concentrations requires the presence of high concentrations of compounds that act as electron donors [76,77]. Based on the results of chemical analysis, the organic electron donors necessary for intensive denitrification are virtually absent. The phosphorus content in the groundwater was below the instrument’s detection limit. The primary cause can be attributed to the development of lithotrophic bacteria that utilize carbonates as a carbon source. The organic matter they produce can subsequently serve as an electron donor for denitrification.

Therefore, the accumulation of organic matter necessary for nitrate removal could be driven by the activity of nitrifying bacteria capable of oxidizing ammonium while utilizing carbonates as a carbon source. However, their active development is likely limited by the low oxygen concentrations [78]. Among ammonium oxidating bacteria (AOB), only *Nitrosomonas* was found in polluted groundwater samples of Sites 1, 3, 4 and 6. Nitrite-Oxidizing Bacteria (NOB) such as *Nitrospira* and *Nitrobacter* were present in polluted Sites 3, 4, 5 and 6 in extremely small quantities (<0.5% OTU abundance). Therefore, the nitrifying family *Nitrosomonadaceae* thrived under these conditions, exhibiting a positive correlation with ammonium levels (r = 0.54, *p* = 0.008). Moreover, ammonium pollution showed strong positive relationships with *Xanthomonadaceae*, *Rhizobiaceae*, *Alcaligenaceae*, and *Microbacteriaceae*. 

Anammox bacteria, detected in samples from Site 2, are capable of participating in the lithotrophic reduction of nitrate using ammonium as an electron donor. Earlier, we demonstrated complete nitrogen removal capacity through the anammox process, with *Brocadiaceae* and *Scalinduaceae* dominating samples 2M and 2H [36,46]. Metagenomic analysis confirmed anammox genetic potential, though only the 2M community (*Ca*. Frigussubterria udmurtiae S40-1) possessed the NrfA nitrite reductase fragment enabling nitrate reduction to ammonium [36]. These analyses also revealed novel taxa, including *Ca*. Frigussubterria, a new *Ca*. Kuenenia species, and two new *Ca*. Scalindua strains. Metagenomic data from Site 2 showed high copy numbers of nitrogen cycle genes (NirC, NarK, NirS, NirK, Hox, Hdh, Nar, NxrABC, NrfA) [36], along with universal stress response capabilities (reactive oxygen species detoxification, assimilatory sulfate reduction) [36] (Figure A2) and outer membrane cytochromes suggesting insoluble Fe(III)/Mn(IV) reduction potential. 

Thus, the nitrogen cycle in the studied aquifers is complex and requires detailed, site-specific investigation. Our analysis identified multiple phylogenetic groups participating in nitrogen removal: denitrifying *Alcaligenaceae* (*Achromobacter*, *Alcaligenes*, *Pusillimonas*) [57,79]; denitrifying and ammonium-oxidizing *Comamonadaceae* (*Acidovorax*, *Comamonas*, *Limnohabitans*, *Rhodoferax*, *Thiomonas*) [69,80]; denitrifying and nitrogen-fixing *Pseudomonadaceae* (*Azotobacter*, *Pseudomonas*, *Rhizobacter*) [69,81]; dissimilatory nitrate-reducing *Hydrogenophilaceae* (*Hydrogenophilus*) [82]; nitrate-reducing *Idiomarinaceae* (*Aliidiomarina*) [56]; nitrogen-fixing and nitrate-reducing *Nocardiaceae* [83,84]; and nitrifying-denitrifying *Sphingomonadaceae* (*Sphingobium*, *Sphingomonas*, *Sphingopyxis*) [85] alongside nitrogen-fixing and denitrifying Xanthomonadaceae (*Arenimonas*, *Luteimonas*, *Lysobacter*, *Thermomonas*, *Xanthomonas)* [85,86]. Moreover, the significant negative correlation between nitrogenous pollutants and OTU richness (Figure A3) suggests a dual role for nitrogen compounds—acting both as stressors and as growth-stimulating nutrients that alleviate natural nitrogen limitation in oligotrophic groundwater systems. 

## Prospects for In Situ Bioremediation

In situ bioremediation of nitrate-contaminated aquifers is a promising remediation strategy. Its implementation is relatively straightforward and requires minimal capital investment. This approach can be performed either by stimulating the intrinsic biogeochemical potential of the autochthonous microbial community through the introduction of soluble organic electron donors, or by supplementing it with specialized microbial consortia (bioaugmentation). The use of such bioaugmentation preparations may be particularly effective in cases of severe pollution. Bioaugmentation has proven to be an effective strategy for nitrate removal across diverse environmental matrices, including wastewater [87], groundwater [88], and soil [89]. 

Our study identified multiple microbial taxa consistently associated with extremal nitrate and ammonium pollution across all sites located over 1000 km apart on average. Comparative analysis revealed a conserved core of bacterial families across all polluted sites, consisting of *Comamonadaceae*, *Gallionellaceae*, *Pseudomonadaceae* (Figure 7), *Sphingomonadaceae*. At the genus level, we identified a universal core of *Acidovorax*, *Pseudomonas*, and *Sphingomonas* across all polluted sites (Figure 8). Furthermore, all highly polluted communities specifically contained *Acinetobacter* and *Limnohabitans*. Most promising candidates for nitrate removal are organotrophic bacteria with modest cultivation requirements, making them suitable for biopreparation development.

Microbial communities in groundwater denitrification hotspots are consistently dominated by denitrifying genera, including *Acidovorax*, *Pseudomonas*, *Sphingomonas*, and *Bacillus,* which harbor the essential functional genes nirS/nirK and nosZ [69,70,76,90,91].

The genus *Pseudomonas*, in particular, is frequently employed as a model denitrifying microorganism due to its well-characterized physiology and genetics [90,92]. It is ubiquitous across diverse ecosystems and is often a dominant taxon within aquifer microbial communities [69,93]. Similarly, the genus *Acidovorax* is a common constituent of groundwater microbiota [70,92]. The presence and activity of these bacterial genera are thus considered critical for the effective natural attenuation of nitrate pollution in groundwater systems [68].

At Site 1, rock-based biofilm enrichment cultures achieved efficient nitrate removal [35,76], yielding pure isolates (*Bacillus proteolyticus* I-16-d, *Paenibacillus glucanolyticus* strains II-2181-a and II-25-e, *Microbacterium flavescens* II-25-m) capable of reducing nitrate, chromate, pertechnetate, and uranyl ions. Previous work also isolated *Shewanella* strains from this site with dual nitrate and uranyl reduction capabilities [94]. The success of this approach, whether using adapted monocultures or specialized bacterial consortia, is largely attributable to the microbial community’s enrichment of key functional genes associated with the nitrate assimilation and Dissimilatory Nitrate Reduction to Ammonium (DNRA) pathways. Among the most promising candidates for such biopreparations are members of the genera *Bacillus* and *Paenibacillus* [76,95].

The utilization of anammox bacteria as bioremediation agents has not yet become widespread, primarily due to the challenges associated with their isolation and cultivation. However, the anammox bacteria discovered in our studies, which inhabit groundwater at a constant temperature of 8–10 °C, are of considerable interest and hold potential for future biotechnology applications.

We successfully established anammox enrichment cultures in flow-through reactors using aquifer-derived biofilms [46]. These enrichments contained not only Planctomycetes but also *Pseudomonadaceae*, *Rhodanobacteraceae*, *Xanthomonadaceae*, and *Alcaligenaceae*. Bioaugmentation trials using Site 2 communities with both native (*Ca*. Scalindua) and non-native (*Ca*. Kuenenia) anammox consortia demonstrated promising results [96].

## 5. Conclusions

Multicomponent groundwater pollution from nitric and sulfuric acid waste storage represents a persistent challenge for mining and ore processing industries. Our comprehensive analysis across six polluted sites identified core microbial taxa—including the families *Comamonadaceae*, *Gallionellaceae*, and *Pseudomonadaceae*—with consistent nitrate reduction capabilities, highlighting their potential for in situ bioaugmentation applications. Field experiments confirm that nitrate removal initiates critical redox transitions that enable subsequent ammonium and sulfate elimination. Our findings demonstrate that pollution chemistry fundamentally shapes aquifer microbial community structure: nitrogenous compounds generally stimulate taxonomic diversity and functional specialization, while sulfate exerts inhibitory effects, significantly reducing biodiversity. The observed decline in microbial diversity and denitrifier abundance in high-nitrate zones indicates limited intrinsic remediation capacity, necessitating bioaugmentation strategies. The dominant microbial groups identified in polluted sites—particularly *Acidovorax*, *Bacillus, Pseudomonas*, *Sphingomonas*, *Acinetobacter*, and *Limnohabitans*—represent optimal candidates for developing targeted biopreparations. Consequently, identifying such widespread microbial taxa across differentially polluted sites provides a robust foundation for developing effective bioremediation consortia for application with organic substrate amendments.

## Figures and Tables

**Table 1:** Sample labeling.

Sample Label	Site	Pollution Level	Depth, m
1B	1. Radioactive Waste Storage Basin “B2”, Siberian Chemical Combine (Seversk, Krasnoyarsk Krai)	Background	15
1M		Medium	15
1H		High	15
2B	2. Slurry Reservoir, Chepetsk Mechanical Plant (Glazov, Udmurt Republic)	Background	11
2M		Medium	11
2H		High	11
3B	3. Slurry Reservoir, Angarsk Electrolysis Chemical Combine (Angarsk, Irkutsk Region)	Background	9
3M		Medium	9
3H		High	9
4B	4. Slurry Reservoir, Electrolysis Chemical Combine (Zelenogorsk, Krasnoyarsk Krai)	Background	12
4M		Medium	12
4H		High	12
5B	5. Slurry Reservoir, Novosibirsk Chemical Concentrates Plant (Novosibirsk, Novosibirsk Region)	Background	15
5M		Medium	15
5H		High	15
6B	6. Slurry Reservoir, Sublimate Plant (Seversk, Krasnoyarsk Krai)	Background	20
6M		Medium	20
6H		High	20

## Data Availability

All data generated or analyzed during this study are included in this published article.

## Appendix A

**Table A1:** Selective media composition.

Bacterial Group	Media Composition
**Aerobic organotrophic bacteria**	10 g/L tryptone, 5 g/L yeast extract, 1 g/L glucose, pH = 7.5.
**Denitrifying bacteria**	Adkins medium for denitrifying bacteria (DB) contained (g/L): NH_4_Cl—1.0; KH_2_PO_4_—0.75; K2HPO4—1.5; NaNO_3_—1.0; NaCl—0.8; Na_2_SO_4_—0.1; MgSO_4_·7H_2_O—0.1; KCl—0.1, yeast extract—0.5; glucose—1.0; CH_3_COONa—1.0, pH = 7, gas phase—Ar.
**Sulfate-reducing bacteria**	Postgate B medium (g/L): NaCl —1.0; KH_2_PO_4_—0.5; NH_4_Cl—1.0; CaSO_4_·2H_2_O—1.0; MgSO_4_·7H_2_O—2; yeast extract—1.0; sodium lactate—4; Na_2_S·9H_2_O—0.2, pH = 7.
**Nitrifying ammonium to nitrite**	Winogradskiy medium for stage I nitrifiers (AOB) contained (g/L tap water): (NH4)_2_SO_4_—2.0; K_2_HPO_4_—1.0; MgSO_4_ ∙ 7H_2_O—0.5; NaCl—2.0; FeSO_4_ ∙ 7H_2_O—0.05; CaCO_3_—5.0, pH = 8.
**Nitrifying nitrite to nitrate**	Winogradskiy medium for stage II nitrifiers (NOB) contained (g/L tap water): NaNO_2_—1.0; K_2_HPO4—0.5; MgSO_4_ ∙ 7H_2_O—0.5; NaCl—0.5; FeSO_4_ ∙ 7H_2_O—0.4; Na_2_CO_3_—1.0, pH = 8.
**Iron reducing bacteria**	Modified IRB medium (g/L tap water): CaCl_2_ ∙ 2H_2_O—0.1; NaH_2_PO_4_—0.6; MgCl_2_ ∙ 6H_2_O—0.1; MgSO_4_ ∙ 7H_2_O—0.1; MnCl_2_ ∙ 4H_2_O—0.005; Na_2_MoO_4_∙ 2H_2_O—0.001; NH_4_Cl—1.0; KCl—0.1; NaCl—0.1; NaHCO_3_—2.0; yeast extract—0.5; CH_3_COONa—0.75; Iron(III) citrate —1.5, pH = 7.

**Table A2:** Sample hydrochemistry, mg/L.

Sample	pH	eH mV	C/C mg/L	NO_3_^−^ mg/L	NO_2_^−^ mg/L	HCO_3_^−^ mg/L	SO_4_^2−^ mg/L	NH_4_^+^ mg/L	Ca^2+^ mg/L	U mg/L	C_org_. mg/L	Na mg/L	Cl mg/L	∑Fe mg/L
1B	7.06	−30	127	<0.20	0.23	79.3	1.8	<0.5	17.5	<0.01	12.2	4.06	1.44	4.8
1M	6.06	65	5070	1500	17.4	241	152	1.7	305	0.06	14.5	245	1.4	4.3
1H	6.56	78	7000	2680	34.5	357	79.3	6.2	308.6	0.72	18.7	639	4.42	17.5
2B	7.38	−105	127	11.5	2.8	53.8	21.4	7.83	13.2	<0.01	3.3	100	10.9	2.1
2M	6.6	78	7900	2240	48.9	1056	2370	361	630.9	0.06	16.9	465	42	4.1
2H	7.4	134	12,800	4300	56.6	1004	970	216	2190	0.43	23.1	2790	2300	5.1
3B	7.39	−115	173	0.8	0.02	126.9	26.7	0.9	33.8	<0.01	3.7	5.25	0.5	13.9
3M	9.26	−50	2700	789	6.9	180.6	974.7	61	461	0.09	4.8	20.6	2.2	0.7
3H	8.03	−3	10,500	3290	52.1	197.6	8248	569	1283	0.04	9.1	117.8	119	0.27
4B	7.7	−273	550	8.54	0.06	267	16	1.21	46	<0.01	13.7	6.21	3.3	0.7
4M	6.41	34	6620	2333.5	48.9	294	142.5	29.6	1143.5	0.12	6	67.4	107	0.6
4H	7.4	80	17,010	11,200	0.06	195	358	1.17	2960	0.14	6.1	137	66	0.9
5B	7.1	−190	729	12.02	1.4	549	30	3.71	123	<0.01	5.5	0.24	11	3.4
5M	7.8	−28	3743	1124	0.6	161	1769	0.23	56	1.58	14.9	685	590	2.6
5H	7.97	90	12,109	6169	44.7	180	470	1.03	1436	0.02	14.6	1249	3200	12.3
6B	7.09	−50	743	0.2	0.5	243.7	12.7	2.4	114.1	<0.01	11.8	6.2	8.6	6.1
6M	7.72	98	2680	209	11.8	358.5	278.2	273.8	24.7	2.04	6.5	42.5	19.2	2.3
6H	7.34	110	1706	121.7	21.9	412.6	290.7	188.9	1188	0.82	12.7	125.7	45.7	2.4
Sample	Standard deviation													
	pH	eH	C/C	NO_3_^−^	NO_2_^−^	HCO_3_^−^	SO_4_^2−^	NH_4_^+^	Ca^2+^	U	C_org_.	Na	Cl	Fe
1B	0.1	−15	11.3	0.0	0.0	5.8	0.1	0.0	1.3	0.0	0.9	0.3	0.1	0.4
1M	0.1	22	386.4	109.5	1.3	17.6	11.1	0.1	22.3	0.0	1.1	17.9	0.1	0.3
1H	0.1	15	442	195.6	2.5	26.1	5.8	0.5	22.5	0.1	1.4	46.6	0.3	1.3
2B	0.11	−23	11.3	0.8	0.2	3.9	1.6	0.6	1.0	0.0	0.2	7.3	0.8	0.2
2M	0.1	14	476.3	163.5	3.6	77.1	173.0	26.4	46.1	0.0	1.2	33.9	3.1	0.3
2H	0.11	25	673.5	313.9	4.1	73.3	70.8	15.8	159.9	0.0	1.7	203.7	167.9	0.4
3B	0.11	−24	23.4	0.1	0.0	9.3	1.9	0.1	2.5	0.0	0.3	0.4	0.0	1.0
3M	0.2	−16	145.8	57.6	0.5	13.2	71.2	4.5	33.7	0.0	0.4	1.5	0.2	0.1
3H	0.15	−5	578	240.2	3.8	14.4	602.1	41.5	93.7	0.0	0.7	8.6	8.7	0.0
4B	0.12	−24	78.3	0.6	0.0	19.5	1.2	0.1	3.4	0.0	1.0	0.5	0.2	0.1
4M	0.1	12	229.5	170.3	3.6	21.5	10.4	2.2	83.5	0.0	0.4	4.9	7.8	0.0
4H	0.11	15	673.5	817.6	0.0	14.2	26.1	0.1	216.1	0.0	0.4	10.0	4.8	0.1
5B	0.1	−21	43.2	0.9	0.1	40.1	2.2	0.3	9.0	0.0	0.4	0.0	0.8	0.2
5M	0.12	−6	165.5	82.1	0.0	11.8	129.1	0.0	4.1	0.1	1.1	50.0	43.1	0.2
5H	0.14	17	567.9	450.3	3.3	13.1	34.3	0.1	104.8	0.0	1.1	91.2	233.6	0.9
6B	0.1	−9	48.8	0.0	0.0	17.8	0.9	0.2	8.3	0.0	0.9	0.5	0.6	0.4
6M	0.12	16	114.1	15.3	0.9	26.2	20.3	20.0	1.8	0.1	0.5	3.1	1.4	0.2
6H	0.11	27	92.6	8.9	1.6	30.1	21.2	13.8	86.7	0.1	0.9	9.2	3.3	0.2

**Table A3:** Multiple ANOVA analysis of main contaminants and responding OTU variance with pollution level groups: background (B), medium (M), high (H).

Parameter	F-Value	*p*-Value	η^2^ (Partial eta-Squared)	Group Difference (Tukey HSD)
**NO_3_^−^**	45.217	2.14 × 10^−9^ *	0.834	B < M < H (*p* < 0.001)
**NH_4_^+^**	62.893	4.78 × 10^−11^ *	0.875	B < M < H (*p* < 0.001)
**SO_4_^2−^**	38.542	1.56 × 10^−8^ *	0.811	B < M, B < H, M ≠ H (*p* < 0.01)
**U**	51.634	5.23 × 10^−10^ *	0.851	B < M < H (*p* < 0.001)
**Fe**	89.326	2.45 × 10^−13^ *	0.908	B < M < H (*p* < 0.001)
**OTU**	28.194	3.67 × 10^−7^ *	0.758	B > M, B > H, M < H (*p* < 0.01)

* *p* < 0.001.

**Table A4:** Two-way ANOVA analysis of site and pollution level variance.

Factor	F-Value	*p*-Value	η^2^ (Partial eta-Squared)
**Site**	28.45	<0.001	0.672
**Contamination level**	45.18	<0.001	0.781
**Site/contamination level**	12.73	<0.001	0.534

**Table A5:** One-way ANOVA analysis of samples within equal pollution level groups: background (B), medium (M), high (H).

Pollution Level	Background	Medium	High
**F-value**	38.72 *	42.15 *	35.89 *

* *p* < 0.001.
